# Facilitation by a Spiny Shrub on a Rhizomatous Clonal Herbaceous in Thicketization-Grassland in Northern China: Increased Soil Resources or Shelter from Herbivores

**DOI:** 10.3389/fpls.2017.00809

**Published:** 2017-05-16

**Authors:** Saixiyala, Ding Yang, Shudong Zhang, Guofang Liu, Xuejun Yang, Zhenying Huang, Xuehua Ye

**Affiliations:** ^1^Inner Mongolia Research Center for Prataculture, State Key Laboratory of Vegetation and Environmental Change, Institute of Botany, Chinese Academy of SciencesBeijing, China; ^2^Inner Mongolia Academy of Agricultural and Animal Husbandry SciencesHohhot, China; ^3^University of Chinese Academy of SciencesBeijing, China

**Keywords:** clonal plant, *Caragana intermedia*, environmental heterogeneity, fertility islands, *Leymus chinensis*, shelter from herbivores, thicketization of grassland

## Abstract

The formation of fertility islands by shrubs increases soil resources heterogeneity in thicketization-grasslands. Clonal plants, especially rhizomatous or stoloniferous clonal plants, can form large clonal networks and use heterogeneously distributed resources effectively. In addition, shrubs, especially spiny shrubs, may also provide herbaceous plants with protection from herbivores, acting as ‘shelters’. The interaction between pre-dominated clonal herbaceous plants and encroaching shrubs remains unclear in thicketization-grassland under grazing pressure. We hypothesized that clonal herbaceous plants can be facilitated by encroached shrubs as a ‘shelter from herbivores’ and/or as an ‘increased soil resources’ under grazing pressure. To test this hypothesis, a total of 60 quadrats were chosen in a thicket-grassland in northern China that was previously dominated by *Leymus chinensis* and was encroached upon by the spiny leguminous plant *Caragana intermedia*. The soil and plant traits beneath and outside the shrub canopies were sampled, investigated and contrasted with an enclosure. The soil organic matter, soil total nitrogen and soil water content were significantly higher in the soil beneath the shrub canopies than in the soil outside the canopies. *L. chinensis* beneath the shrub canopies had significantly higher plant height, single shoot biomass, leaf length and width than outside the shrub canopies. There were no significantly differences between plant growth in enclosure and outside the shrub canopies. These results suggested that under grazing pressure in a grassland undergoing thicketization, the growth of the rhizomatous clonal herbaceous plant *L. chinensis* was facilitated by the spiny shrub *C. intermedia* as a ‘shelter from herbivores’ more than through ‘increased soil resources’. We propose that future studies should focus on the community- and ecosystem-level impacts of plant clonality.

## Introduction

The encroachment of woody plants into grasslands, or the thicketization of grasslands, occurs worldwide (Archer, 1995; van Auken, 2000; Asner et al., 2003; Cabral et al., 2003; Laiolo et al., 2004; Brook and Bowman, 2006; Wigley et al., 2010; Soliveres et al., 2014; Masson et al., 2015). Possible drivers of these changes in the vegetation structure include climate change (Fensham et al., 2005; IPCC, 2013), livestock grazing (Brown and Archer, 1999; Sharpe and Bowman, 2004), altered fire regimes (Brook and Bowman, 2006; Srinivasan, 2012), and elevated carbon dioxide (Bond and Midgley, 2000). Through an extensive shift in the plant community structure, the thicketization of grasslands has strong potential to alter key ecosystem processes (Zavaleta and Kettley, 2006). It may lead to a decline in biodiversity (Báez and Collins, 2008), a reduction in ecosystem functioning and resilience (Caldeira et al., 2015), a loss of ecosystem carbon (Jackson et al., 2002), an increase in the soil quality (Mills and Fey, 2004; Liao et al., 2006), an increase in the soil microbial biomass (Liao and Boutton, 2008) or the enhancement of soil animal activity (Hobbs and Mooney, 1986; Fabricius et al., 2003). Soliveres et al. (2014) found that plant diversity and multifunctionality peaked at intermediate levels of woody cover. However, another 15-year study showed that the shrub invasion in an undisturbed wetland had few community-level effects (Mills et al., 2009). McKinley et al. (2008) also reported that the conversion of grasslands to coniferous woodland has a limited effect on soil nitrogen cycle processes. Anyway, it is commonly recognized that the thicketization of grassland may increase spatial heterogeneity in soil resources and enhance soil nutrient levels on small spatial scales due to the formation of fertility islands by shrubs in arid, semi-arid, and semi-humid areas (Rong et al., 2016).

Soil resources are enriched under shrub canopies, forming so-called ‘fertility islands’ (Schlesinger et al., 1996), which might affect seedling establishment (Maestre et al., 2003), plant–plant interactions (Aguiar and Sala, 1999), species distribution (Pan et al., 1998), the diversity and productivity of plant communities (Mou et al., 1995; Anderson et al., 2004), microbial activity/diversity (Molina-Montenegro et al., 2016), and the abundance and diversity of mycorrhizal fungi (Casanova-Katny et al., 2011). Many studies have documented higher nutrient levels in fertility island soil (Ludwig and Tongway, 1997; Bochet et al., 1999; Hirobe et al., 2001; Li et al., 2007, 2008). Higher nutrient levels and higher competition from shrub common affects the growth of grass beneath shrub canopies, while lower nutrient levels and lower competition from shrub affects the growth of grass outside shrub canopies. The ultimate effects (positive or negative) of fertility islands on the growth of grass were species-specific (Zhang et al., 2016).

Clonal plants, especially rhizomatous or stoloniferous clonal plants, occupy relatively large habitats (Liu et al., 2007), dominate many ecosystems (Song et al., 2002), and play a key role in thicketization-grassland (Formica et al., 2014). Clonal plants can efficiently use heterogeneously distributed resources through their unique features, such as clonal plasticity (Gough et al., 2012), clonal integration (Roiloa and Hutchings, 2013; Liu et al., 2016), the intra-clonal division of labor (de Kroon and Hutchings, 1995), and clonal foraging (Yan et al., 2013), although there are trade-offs between different clonal growth forms (Ye et al., 2006, 2015). Clonal plants have the potential to decrease the differences in resource supplies between different parts of the clones (Eriksson and Jerling, 1990), and then may have powerful effects on resource heterogeneity (Magyar et al., 2004; Cornelissen et al., 2014). Rhizomatous or stoloniferous clonal plants always create a clonal network consisting of a large number of interconnected ramets (Charpentier et al., 2012; Song et al., 2013), some of which are distributed beneath shrub canopies in thicketization-grassland. A large body of evidence has demonstrated that ramets growing in high-resource patches translocate resources to the interconnected ramets growing in low-resource patches through horizontal structures such as rhizomes, stolons, or roots (Atkinson and Else, 2012; Roiloa and Hutchings, 2013; Pinno and Wilson, 2014; Roiloa et al., 2014). That observation indicates that the rich resources in the soil of fertility islands might be translocated by ramets that are distributed beneath shrub canopies to ramets that are distributed in the interspace between shrubs to promote the growth of ramets outside the canopy, resulting the feedback of soil nutrient outside the canopy through plant decomposition (Magyar et al., 2004) and/or resources releasing (Ye et al., 2016). Thus, when shrubs encroach into an ecosystem dominated by rhizomatous clonal plants, the effects of shrub fertility islands may then diminish via the influence of clonal plants.

Shrubs with poor palatability, especially spiny shrubs, may provide herbaceous plants with protection from herbivores by reducing access to plants living underneath their canopies (Rousset and Lepart, 1999). Under grazing pressure, clonal herbaceous plants may make the shrub canopies as ‘shelter from herbivores’ and selectively distribute more resources (higher plant biomass and/or higher ramets number) beneath the canopies. Additionally, the decaying biomass of clonal plants feeds nutrients back into the soil (Magyar et al., 2004). Thus, clonal plants can intensify the effects of shrub fertility islands.

The aim of the present study is to investigate the effects of a spiny shrub, *Caragana intermedia*, on a rhizomatous herbaceous plant, *Leymus chinensis*, in the thicketization-grassland of Inner Mongolia, China. We hypothesized that clonal plants can be facilitated by encroached shrubs as a ‘shelter from herbivores’ and/or as an ‘increased soil resources’ under grazing pressure. Specifically, does a clonal plant network of *L. chinensis* diminish or intensify the effects of the fertility islands formed by *C. intermedia* shrubs? Alternatively, does the clonal herbaceous zone treat the presence of the shrub canopy as a ‘increased soil resources’ more than a ‘shelter from herbivores’?

## Materials and Methods

### Study Site

This study was conducted in Siziwang Banner Research Station, affiliated with the Inner Mongolia Academy of Agriculture and Animal Husbandry Sciences. The station (41°47.28′ N, 111°53.77′ E; 1450 m, a. s. l.) is located in western Inner Mongolia, China, and it has a temperate continental climate that is characterized by a short growing season and a long, cold winter. The mean annual temperature ranges from 5.0 to 8.5°C, with a minimum mean month temperature of -15.1°C (January) and a maximum mean month temperature of 19.6°C (July). The mean annual precipitation is 280 mm, with most precipitation occurring between June and September.

Desert grassland is the dominant ecosystem in this area, and it is dominated by *Stipa breviflora* Griseb. (Gramineae), *Artemisia frigida* Willd. (Asteraceae), and *Cleistogenes songorica* (Roshev.) Ohwi (Gramineae), accompanied by *Convolvulus ammannii* Desr. (Convolvulaceae), *Heteropappus altaicus* (Willd.) Novopokr. (Asteraceae), *C. stenophylla* Pojark. (Fabaceae), *C. intermedia* Kuang et H. C. Fu (Fabaceae), and *L. chinensis* (Trin.) Tzvelev (Gramineae). However, the thicketization of grassland occurs frequently due to the long-term overgrazing (about 1 sheep per hectare in summer) in this area (Zhao et al., 2014; Supplementary Figure 1). In our study site, the encroaching spiny shrub *C. intermedia* has poor palatability, and the rhizomatous clonal plant *L. chinensis* has strong clonal growth and dominates the thicketization-grassland. *L. chinensis* always form a large rhizomatous network which distributes in the 5–15 cm soil layer, and the rhizomatous between ramets can survive for approximately 4 years (Bai et al., 2009).

### Field Sampling and Laboratory Analysis

In September 2014, a 200 × 200 m *C. intermedia*–*L. chinensis* plant community near the station was chosen. Forty-eight shrub canopies of *C. intermedia* with different size (from 60 cm × 50 cm to 500 cm × 358 cm) was chose and the shrub crown width (both the long and narrow sides) was measured. A 50 cm × 50 cm quadrats was established in the center of each shrub canopy for field investigation and sampling. For contrast, six 50 cm × 50 cm quadrats outside the *C. intermedia* canopies in the community and six 50 cm × 50 cm quadrats in an enclosure (an area of 4 ha, southwest 600 m of investigated community) were also chosen for field investigation and sampling. This enclosure was dominated by *L. chinensis* but without shrub encroachment, and in which grazing had been prohibited for more than 10 years. For each quadrat, the coverage and abundance of *L. chinensis* were measured, and five plants (that had not been damaged by animals) were chosen for measuring plant height; five intact leaves per plant were chosen for measuring the leaf length and width. All the living shoot biomass of *L. chinensis* was harvested and oven-dried at 75°C for ≥24 h to a constant mass before being weighed. A soil core (5 cm in diameter) was taken and divided into two strata from the 0–10 cm and 10–20 cm depths. Half of each soil sample was weighed to obtain the fresh weight, followed by oven-drying at 150°C for ≥48 h and weighing to obtain the dry weight, and then calculated soil water content (SWC). The other half was used for chemical analysis. For each quadrat beneath the *C. intermedia* canopies, the coverage, plant height and branch density of *C. intermedia* were also measured.

The soil total nitrogen (STN) and soil total phosphorus (STP) were analyzed according to the micro-Kjeldahl method (Kjeltec 2200 Auto Distillation Unit, FOSS, Sweden), the soil organic matter (SOM) was analyzed using an elemental analyzer (Vario EL III, CHNOS Elemental Analyzer, Elementar Analysensysteme GmbH, Germany), and the pH was measured by soil acidometer titration (Sartorius PB-10, Sartorius, Germany).

### Data Analysis

We calculated the single shoot biomass of *L. chinensis* by taking the total shoot biomass divided by the number of individuals. Statistical analyses were performed using SPSS18 (SPSS Inc., United States, 2009). A One-way analysis of variance (ANOVA) was used to test the difference in plant and soil traits among different sites (beneath *C. intermedia* canopies under grazing, outside *C. intermedia* canopies under grazing, and under grazing prohibition), followed by Tukey’s HSD tests for multiple comparisons. We used two-way ANOVAs to analyze the effects of different soil layers and different sites on soil traits. And a two-tailed Pearson’s correlation test was used to analyze the relationship between each *L. chinensis* and *C. intermedia* trait. We also used linear regressing to analysis the relationship between plant cover of *C. intermedia* and plant cover, plant density and plant biomass of *L. chinensis* beneath the shrub canopies. The data for the *L. chinensis* coverage, soil pH and STN were log_10_ (*x*+1)-transformed to satisfy the requirements for normality and homogeneity of variance.

## Results

**FIGURE 1:**
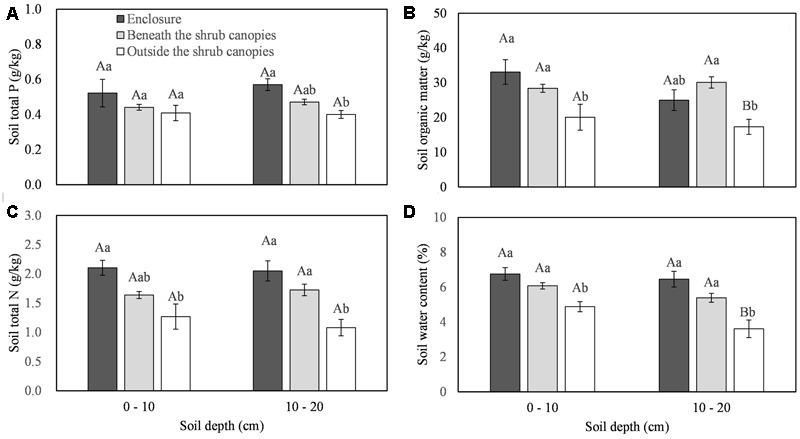
**Effects of the sampling sites (enclosure, beneath the shrub canopies, and outside the shrub canopies) on the soil total P (A)**, soil organic matter **(B)**, soil total N **(C)** and soil water content **(D)** in different soil layers. Different capital letters indicate a significant difference between the 0–10 cm and 10–20 cm soil layers, and different lowercase letters indicate a significant difference among the three sampling sites at *P* < 0.05.

All the soil traits showed statistically significant differences among the different types of sites (beneath *C. intermedia* canopies under grazing, outside *C. intermedia* canopies under grazing, and under grazing prohibition), except for STP at the 0–10 cm depth (**Table 1**). Soil outside the *C. intermedia* canopies had the lowest SOM, STN, and SWC at both the 0–10 cm and 10–20 cm depths, and the lowest STP at the 10–20 cm depth (**Figure 1**). In comparison with the soil beneath the *C. intermedia* canopies, the soil outside the canopies had significantly lower SOM (Tukey’s test, *P* = 0.046) and marginally significantly lower SWC (Tukey’s test, *P* = 0.050) at the 0–10 depth and lower SOM (Tukey’s test, *P* = 0.019), SWC (Tukey’s test, *P* = 0.047), and STN (Tukey’s test, *P* = 0.042) at the 10–20 cm depth (**Figure 1**). Compared with the soil under grazing prohibition, the soil outside the *C. intermedia* canopies under grazing had significantly lower STN (Tukey’s test, *P* = 0.003), SOM (Tukey’s test, *P* = 0.020), and SWC (Tukey’s test, *P* = 0.023) at the 0–10 cm depth and lower STN (Tukey’s test, *P* = 0.031), SWC (Tukey’s test, *P* = 0.013), and STP (Tukey’s test, *P* = 0.018) at the 10–20 cm depth (**Figure 1**). There were no statistically significant differences in the soil traits between the soil beneath the canopies and the soil under grazing prohibition (**Figure 1**).

**Table 1:** Effects of the sample sites (enclosure, beneath the shrub canopies, and outside the canopies) on soil characteristics and the clonal herbaceous plant *Leymus chinensis*.

	df	*F*	*P*
**Soil**			
Soil organic matter (0–10 cm)	2, 56	4.071	**0.022**
Soil organic matter (10–20 cm)	2, 56	4.218	**0.020**
Soil pH (0–10 cm)	2, 56	23.142	**<0.001**
Soil pH (10–20 cm)	2, 56	9.701	**<0.001**
Soil water content (0–10 cm)	2, 56	3.944	**0.025**
Soil water content (10–20 cm)	2, 56	4.508	**0.015**
Soil total phosphorus (0–10 cm)	2, 56	1.418	0.251
Soil total phosphorus (10–20 cm)	2, 56	4.042	**0.023**
Soil total nitrogen (0–10 cm)	2, 56	5.949	**0.005**
Soil total nitrogen (10–20 cm)	2, 56	3.693	**0.031**
***Leymus chinensis***			
Plant density	2, 56	1.316	0.276
Plant cover	2, 56	2.690	**0.076**
Plant height	2, 56	5.420	**0.007**
Plant biomass	2, 56	0.194	0.824
Leaf length	2, 56	3.474	**0.038**
Leaf width	2, 56	9.381	**<0.001**
Single plant biomass	2, 56	5	**0.009**

*Bold type indicates significant effects at *P* < 0.05.*

**FIGURE 2:**
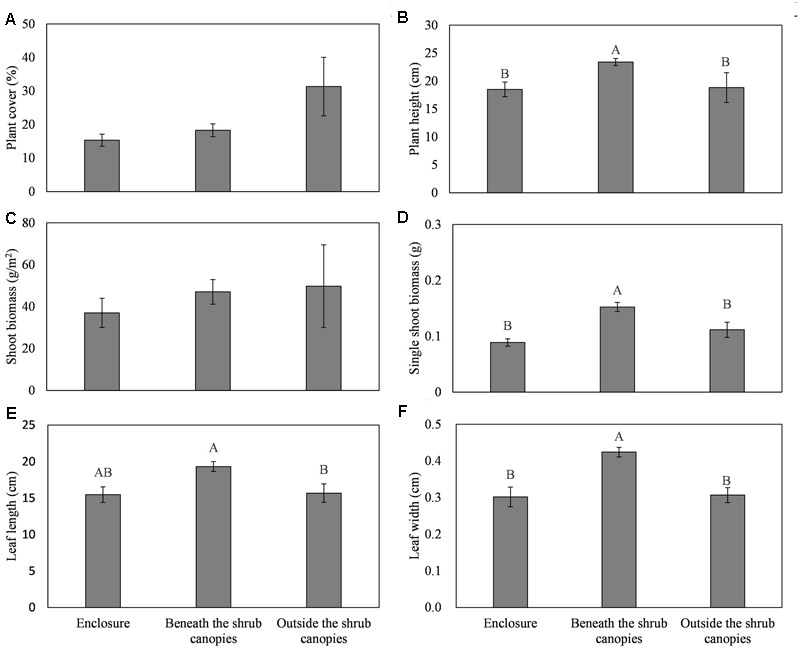
**Effects of the sampling sites (enclosure, beneath the shrub canopies, and outside the shrub canopies) on the plant cover (A)**, height **(B)**, shoot biomass **(C)**, single shoot biomass **(D)**, leaf length **(E)**, and leaf width **(F)** of the clonal herbaceous plant *L. chinensis*. Different letters represent significant differences among the three sampling sites, while the same letters and/or no letters indicate no significant differences between any treatments at *P* < 0.05.

The site significantly affected the plant cover, plant height, leaf length and width, and single-plant biomass of *L. chinensis*. However, there was no significant effect of the site on the plant density and plant biomass (**Table 1**). The *L. chinensis* beneath the *C. intermedia* canopies had the highest values for plant height, single shoot biomass, and leaf length and width, and showed similar plant cover and shoot biomass among the three sampling sites (**Figure 2**). There was no significant difference in the plant traits of *L. chinensis* between the area under grazing prohibition and outside the shrub canopies under grazing (**Figure 2**).

**FIGURE 3:**
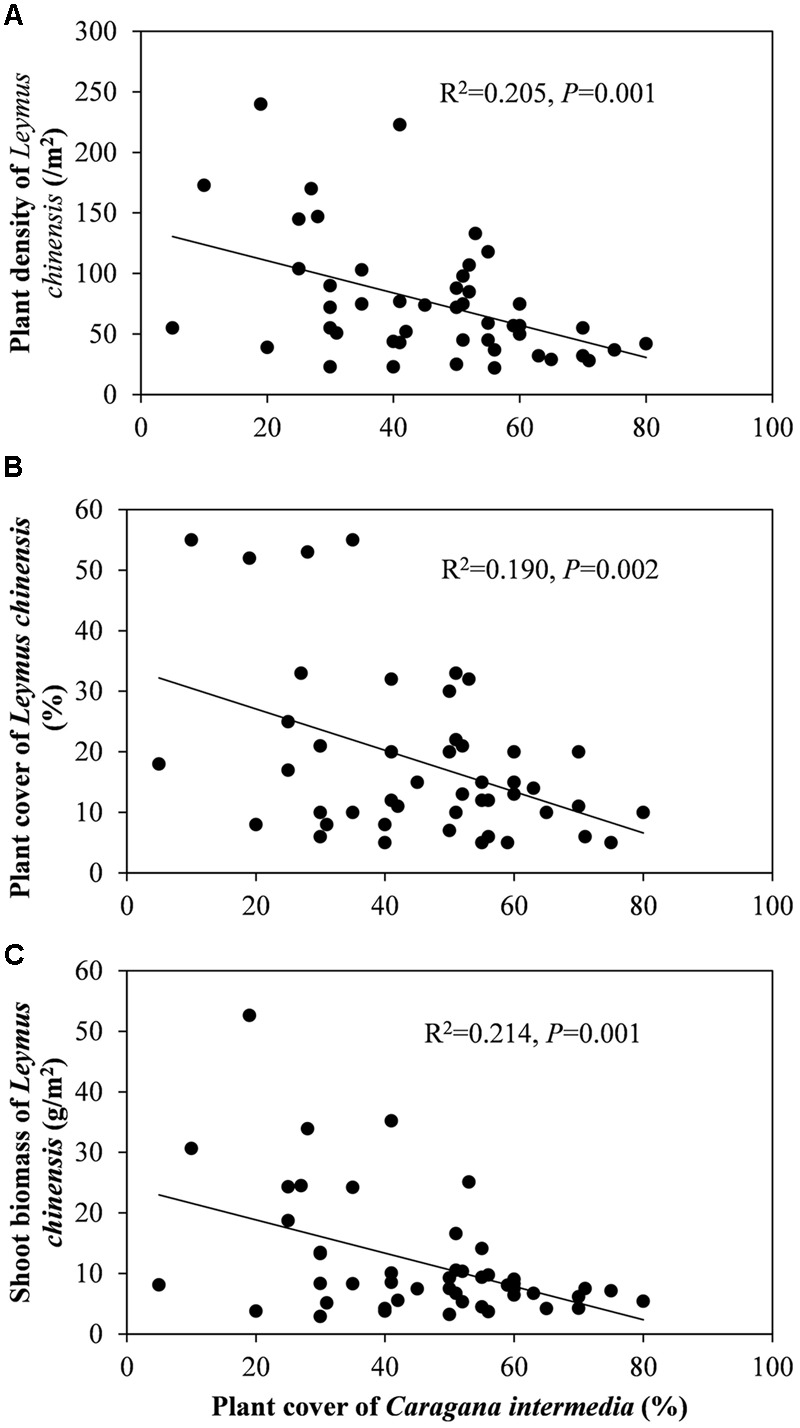
**Relationship between plant cover of *Caragana intermedia* and plant density (A)**, plant cover **(B)** and plant shoot biomass **(C)** of *Leymus chinensis* beneath the canopies of *C. intermedia*.

Beneath the *C. intermedia* canopies, the plant density, cover and biomass of *L. chinensis* showed significantly negative relationships with the plant cover of *C. intermedia*, and the leaf length of *L. chinensis* had a significantly positive relationship with the crown width of *C. intermedia* (**Table 2** and **Figure 3**). There were no significant relationships between other *L. chinensis* and *C. intermedia* traits (**Table 2**).

**Table 2:** The relationships between characteristics of the shrub species *Caragana intermedia* and the clonal herbaceous plant *Leymus chinensis*.

*Leymus chinensis*		*Caragana intermedia*				
		Density of branches	Plant cover	Crown width (long)	Crown width (narrow)	Plant height
Plant density	Pearson correlation	-0.194	-0.453	-0.186	-0.175	0.149
	Sig. (2-tailed)	0.186	**0.001**	0.205	0.234	0.312
Plant cover	Pearson correlation	-0.199	-0.436	-0.103	-0.049	-0.023
	Sig. (2-tailed)	0.174	**0.002**	0.484	0.742	0.877
Plant height	Pearson correlation	-0.130	-0.219	-0.041	0.015	0.060
	Sig. (2-tailed)	0.380	0.134	0.784	0.920	0.683
Plant biomass	Pearson correlation	-0.247	-0.462	-0.187	-0.149	0.088
	Sig. (2-tailed)	0.091	**0.001**	0.204	0.311	0.550
Single plant biomass	Pearson correlation	-0.181	-0.056	-0.057	-0.004	-0.151
	Sig. (2-tailed)	0.219	0.707	0.703	0.979	0.307
Leaf length	Pearson correlation	-0.199	-0.166	0.296	0.323	-0.236
	Sig. (2-tailed)	0.174	0.260	**0.041**	**0.025**	0.106
Leaf width	Pearson correlation	-0.087	-0.174	0.010	0.063	0.010
	Sig. (2-tailed)	0.555	0.237	0.946	0.670	0.944

*Bold type indicates significant effects at *P* < 0.05.*

## Discussion

Positive interactions among plants, or facilitation, can occur when the presence of one plant enhances the growth, survival, or reproduction of a neighbor. Canopy facilitation plays a key role in the interaction between trees or shrubs and grass in thicketization-grassland, which is very common in semiarid and desert grasslands (Scholes and Archer, 1997; van Auken, 2000; Callaway, 2007). In northern China, livestock grazing has been demonstrated as a primary factor in the thicketization of grassland (Zhao et al., 2014). Our results showed that under grazing pressure, the rhizomatous grass *L. chinensis* growth was facilitated by *C. intermedia* shrub in northern China, with the highest plant performance occurring beneath the *C. intermedia* canopy (**Table 1** and **Figure 2**). This canopy facilitation may be due to direct mechanisms such as fertility islands and/or to indirect mechanisms such as herbivore-mediated control (Callaway, 2007 as a review).

Higher levels of nutrients, in forms such as higher SOM and/or STN, were found in the soil beneath the shrub canopy in sandy grassland (Hirobe et al., 2001; Li et al., 2008), typical steppe (Peng et al., 2014), desert steppe (Pan et al., 2015), desert (Schlesinger et al., 1996; Li et al., 2007) and savanna (Mills and Fey, 2004), due to the effects of fertility islands (Schlesinger et al., 1996). A meta-analysis showed that shrub encroachment would increase the soil organic carbon content in semi-arid and humid regions, and there was a greater rate of increase in grassland that was encroached upon by leguminous shrubs than in grassland encroached upon by non-legumes (Li et al., 2016). In our study, significantly higher SOM, STN, and marginally significantly higher SWC were found in the soil beneath shrub canopies than in the soil outside the canopies (**Table 1** and **Figure 1**), indicating that fertility islands were actually formed during the thicketization of grassland that was previously dominated by the rhizomatous clonal plant *L. chinensis*. If we do not consider the clonal plant traits, it seems that *L. chinensis* growth was facilitated by the effects of *C. intermedia* fertility islands in our study.

Rhizomatous clonal plants, such as *L. chinensis* in our study, always create large clonal networks consisting of a large number of interconnected ramets (Zhou et al., 2015), and they may cover a number of shrub canopies in the thicketization-grassland. These plants can flexibly regulate their biomass allocation to cope with changing resource availability (Xie et al., 2016). Considering the greater soil resources and lower light conditions beneath the shrub canopies, clonal plants may selectively distribute more ramets beneath the shrub canopies, and they may allocate more biomass to the roots of ramets beneath the canopies to absorb the ‘rich’ soil resources beneath shrub canopies effectively and then transport these resources to ramets outside the canopies through clonal integration (Ye et al., 2015). These resources can be used by clonal plants themselves, or they can even be redistributed into the soil and used by other plants outside the shrub canopies (Cornelissen et al., 2014; Ye et al., 2016). Therefore, one hypothesis is that the *L. chinensis* plants beneath the shrub canopies may have a higher plant density and lower shoot biomass/higher root biomass, and the difference in soil quality between the areas beneath and outside the *C. intermedia* canopies may be diminished in the context of our experiment. However, this hypothesis was obviously inconsistent with our results. Our results showed that *L. chinensis* allocated more resources to the shoots of the ramets beneath the shrub canopies to enhance their competitive ability and to then insure the growth of the whole clonal network under grazing pressure. In our study, clonal plant *L. chinensis* seems to benefit from *C. intermedia* shrub as a ‘shelter from herbivores’ more than as a source of ‘increased soil resources.’

Grazing is one of the primary causes for the scarcity of native herbs between the bushes and the relative abundance beneath the bushes (Jaksic and Fuentes, 1980). Under grazing pressure, shrubs may provide the herbaceous plants beneath their canopies with protection from herbivores (Rousset and Lepart, 1999), an even more important factor than mitigating abiotic stress (Louthan et al., 2014). Clonal plants may place their ramets in different microhabitats to spread the mortality of the organism and to engage in so-called risk-spreading (Eriksson and Jerling, 1990; Dong, 1996). To spread the risk from herbivores, *L. chinensis* in our experiment distributed some ramets beneath the *C. intermedia* canopies. *L. chinensis* ramets beneath the shrub canopies showed higher shoot biomass and larger leaves, allowing them to adapt to the lower light and higher inter-species competition (**Figures 1, 2**). The decaying biomass of clonal plants feeds nutrients back into the soil (Magyar et al., 2004) and then enhances the effects of shrub fertility islands, to some extent, leading to higher SOM, STN, and SWC in our study.

The interactions between shrubs and herbaceous plants are complex, including bidirectional positive and negative effects, and the ratios between positive and negative effects can shift (Holzapfel and Mahall, 1999). Facelli and Temby (2002) found that shrubs can have simultaneously facilitative and inhibitory effects on the annual plants through different mechanisms, and more importantly, different shrub species have different effects. We found that *C. intermedia* provided *L. chinensis* with facilitation (positive effects) relating to ‘shelter from herbivores’ and/or ‘increased soil resources.’ However, we also found a significantly negative relationship between the *C. intermedia* plant cover and plant density and the cover and biomass of *L. chinensis*, indicating some level of competition (negative effects) between these two species (**Table 2** and **Figure 3**). The ‘stress gradient hypothesis’ predicts that competition should predominate in low-stress environments, with facilitation increasing in strength and/or frequency in high-stress areas (Bertness and Callaway, 1994; Callaway et al., 2002). This model indicates that if the grazing pressure is weakened to less than the competition pressure from the shrubs, or even eliminated, then the relationship between shrubs and clonal plants may change. Without grazing pressure, the clonal plants may allocate more biomass to the roots of the ramets beneath the shrub canopy to absorb the ‘rich’ soil resources and then diminish the effect of the fertility islands formed by shrubs. A previous study showed that the changes in the direction and magnitude of the effects of shrub encroachment on soil varied with abiotic (climate and soil) and biotic (shrub species) factors (Li et al., 2016). Clonal integration may be one of the factors affecting the direction and magnitude of the variation in soil traits during the encroachment of shrubs into grassland since this integration can diminish or enhance the effect of the fertility islands formed by shrubs.

Plant clonality plays a key role in the thicketization of grassland, and many studies have focused on the encroachment of clonal shrubs into grassland (Lett and Knapp, 2005; Smit et al., 2010). Formica et al. (2014) found that clonal growth (78%) accounts for more woody plant expansion than seed dispersal (22%). However, there have been few studies on the encroachment of woody plants into grassland dominated by clonal plants (Kesting et al., 2015; Zhang et al., 2016). Our study provides novel and firm evidence to support the idea that the growth of the rhizomatous grass *L. chinensis* was facilitated by the canopy of the *C. intermedia* shrub, and this facilitation may be due to ‘shelter from herbivores’ more than to ‘increased soil resources’ under grazing pressure in northern China. These results can help with understanding the process and ecological consequences of woody plant encroachment into grasslands that are dominated by clonal plants.

We believe that the process and ecological consequences of woody plant encroachment into grasslands that are dominated by clonal plants may be different from those in grasslands dominated by non-clonal plants, due to the unique features of clonal plants. We also acknowledge that the present study was based on a rough field investigation, and it may not completely explain the effects of clonal plants during the thicketization of grassland. More precision and empirical experiments are needed to confirm the interactions between clonal plants and shrubs, and long-term experiments are also needed to focus on the community- and ecosystem-level impacts of plant clonality, with or without disturbances such as grazing.

## Conclusion

The formation of fertility islands by the leguminous shrub *C. intermedia* increased the soil resources beneath the shrub canopies and provided the clonal herbaceous *L. chinensis* beneath the shrub canopies with protection from herbivores in thicketization-grassland in northern China. Under grazing pressure, the growth of the clonal plant *L. chinensis* beneath the shrub canopy was facilitated by the spiny shrub *C. intermedia* as a ‘shelter from herbivores’ more than as an ‘increased soil resources’ in a thicketization-grassland that was previously dominated by the rhizomatous clonal plant *L. chinensis*. These results can help to understand the process and ecological consequences of woody plant encroachment into grasslands dominated by clonal plants. We propose that future studies should focus on the community- and ecosystem-level impacts of plant clonality.

## Author Contributions

XHY directed, coordinated, and funded this study with intellectual input from ZH. S, DY, XHY, GL, and XJY carried out the fieldwork and lab analyses. XHY, S, and SZ did the data analysis and wrote the first manuscript draft. All authors commented on the manuscript and consent with the submitted version.

## Conflict of Interest Statement

The authors declare that the research was conducted in the absence of any commercial or financial relationships that could be construed as a potential conflict of interest.
