# Supplementary material for: Facilitation by a Spiny Shrub on a Rhizomatous Clonal Herbaceous in Thicketization-Grassland in Northern China: Increased Soil Resources or Shelter from Herbivores
Source: Front Plant Sci. 2017 May 16;8:809. doi: 10.3389/fpls.2017.00809 (PMC5432564; doi:10.3389/fpls.2017.00809)

Supplementary Information

Facilitation by a spiny shrub on a rhizomatous clonal herbaceous in thicketization-grassland in northern China: increased soil resources or shelter from herbivores

Saixiyala[1, 2], Ding Yang [1, 2], Shudong Zhang[1,3], Guofang Liu[1], Xuejun Yang [1], Zhenying Huang[1,*], Xuehua Ye[1,*]

[*]Correspondence author: Zhenying Huang (zhenying@ibcas.ac.cn); Xuehua Ye (yexuehua@ibcas.ac.cn)

Supplementary figure 1. Photo of thicketzation-grassland with the pressure of grazing (A), and ramets of clonal herbaceous plant *Leymus chinensis* beneath the canopy of spiny shrub *Caragana intermedia* (B).

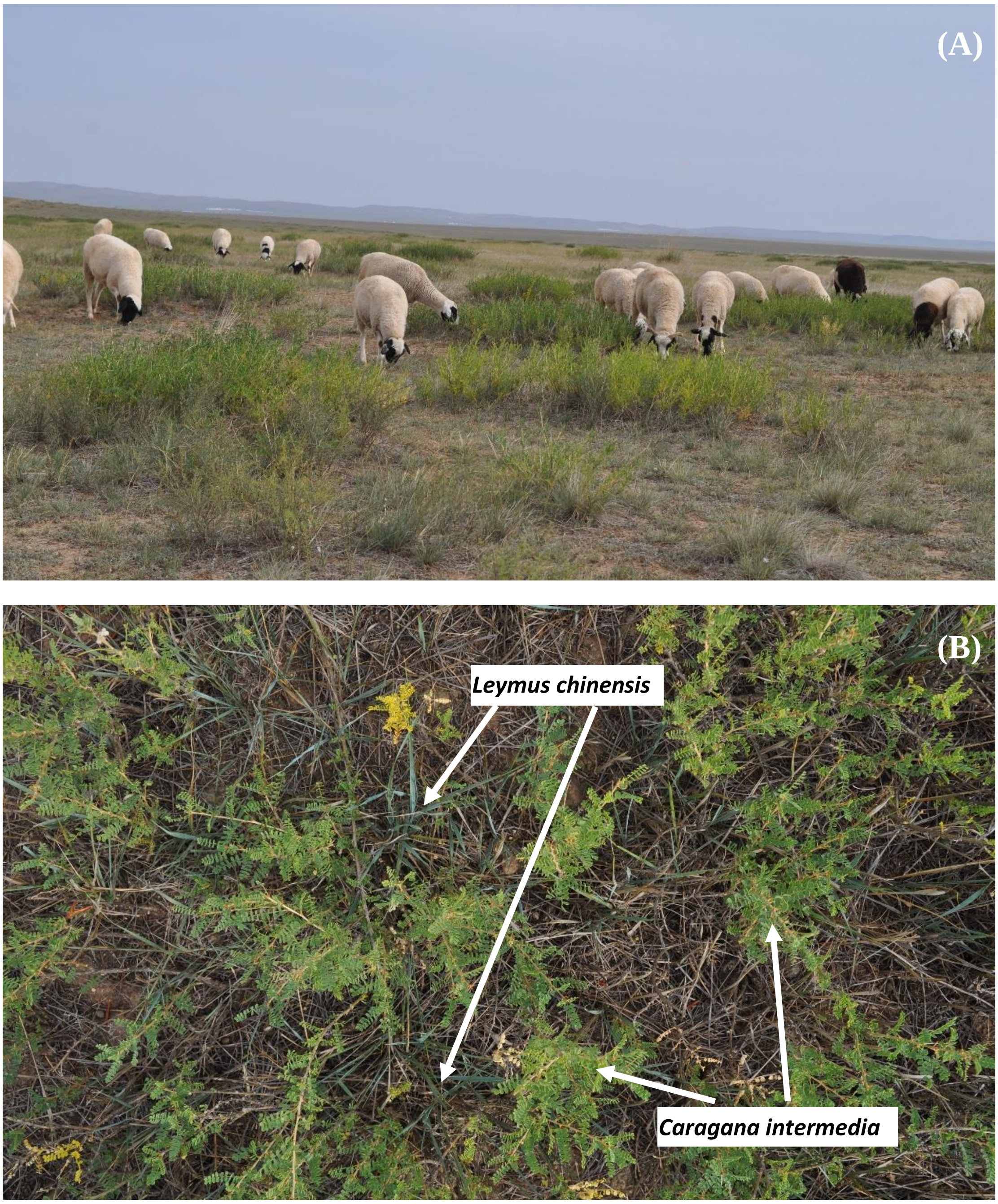

Supplement: Supplementary file 1 [file Image_1.PDF]
